# Pro-Angiogenic Effects of Chalcone Derivatives in Zebrafish Embryos *in Vivo*

**DOI:** 10.3390/molecules200712512

**Published:** 2015-07-09

**Authors:** Yau-Hung Chen, Chao-Yuan Chang, Chiung-Fang Chang, Po-Chih Chen, Ya-Ting Lee, Ching-Yuh Chern, Jen-Ning Tsai

**Affiliations:** 1 Department of Chemistry, Tamkang University, 151, Ying-chuan Road, Danshui District, New Taipei City 25137, Taiwan; E-Mails: frank00634@hotmail.com (C.-Y.C.); joanna20520@gmail.com (Y.-T.L.); 2 Department of Life Sciences, National Chung Hsing University, Taichung 40227, Taiwan; E-Mail: kitomint@hotmail.com; 3 Department of Applied Chemistry, National Chia-Yi University, Chia-Yi 60004, Taiwan; E-Mail: tpdyha6606@gmail.com; 4 School of Medical Laboratory and Biotechnology, Chung Shan Medical University, Taichung 40201, Taiwan; 5 Clinical Laboratory, Chung Shan Medical University Hospital, Taichung 40201, Taiwan

**Keywords:** angiogenesis, chalcones, *in vivo*, zebrafish embryos

## Abstract

The aim of this study was to investigate novel chalcones with potent angiogenic activities *in vivo*. Chalcone-based derivatives were evaluated using a transgenic zebrafish line with fluorescent vessels to real-time monitor the effect on angiogenesis. Results showed that the chalcone analogues did not possess anti-angiogenic effect on zebrafish vasculatures; instead, some of them displayed potent pro-angiogenic effects on the formation of the sub-intestinal vein. Similar pro-angiogenic effects can also be seen on wild type zebrafish embryos. Moreover, the expression of *vegfa*, the major regulator for angiogenesis, was also upregulated in their treatment. Taken together, we have synthesized and identified a series of novel chalcone-based derivatives as potent *in vivo* pro-angiogenic compounds*.* These novel compounds hold potential for therapeutic angiogenesis.

## 1. Introduction

Chalcone (1,3-diphenyl-2-propen-1-one), abundant in edible plants, is essential for the biosynthesis of flavonoids and isoflavonoids [1]. Natural occurring chalcones and chalcone-based derivatives have been demonstrated to possess diverse pharmacological activities, such as anti-oxidant, anti-inflammatory, anti-bacterial, and anti-tumor activity [2,3,4,5,6,7]. A recent study indicated that one of the mechanisms for anti-tumor activity is to inhibit angiogenesis [8].

Angiogenesis, the formation of new blood vessels from pre-existing vessels, is required for various physiological processes, such as development, growth and wound healing [9]. Angiogenesis occurs during embryonic development, and continues into adult life. Many human diseases arise from inadequate changes in tissue vascularization and oxygen availability [10]. For example, insufficient vascular growth contributes to coronary artery disease, while excess angiogenesis promotes growth and metastasis of tumor. Therefore molecules possessing pro- or anti-angiogenic effect hold great potential for treating diseases. During normal physiological condition, the balance between pro- and anti-angiogenic factors is essential for regulating angiogenesis [11]. VEGF and its tyrosine kinase receptors (VEGFRs) are the key regulators in angiogenesis and are highly conserved across vertebrate species [12]. Among them, VEGF-A, the most important member of VEGF, binds and activates the VEGFR2 (KDR), subsequently activate the main signaling pathway [13].

Many *in vitro* and *in vivo* models have been used for analyzing angiogenesis. These include the endothelial cell line derived from human umbilical cord vein or from organ specific endothelial cells. Many *in vivo* models, such as chick embryo, rabbit and mouse, have also been developed for analyzing angiogenesis [14]. However, large-scale chemical screening with these models has been hampered due to the cost and space needed for breeding. Zebrafish (*Danio* *rerio*) is a useful tool for analyzing developmental processes and modeling human genetic diseases [15]. Zebrafish possess a closed circulatory system, and the molecular mechanisms underlying its vascular development are highly similar to those of higher vertebrates [16]. Compared with other animal models, zebrafish offers several advantages in studying vascular development. For example, their embryos are transparent during early development and they can survive through the first week with no blood circulation. Finally, the generation of the transgenic zebrafish fish line with fluorescent vessels, such as Tg(*fli1:egfp*) [17], allows real-time observation of vascular growth *in vivo*. These features have made zebrafish a powerful animal model in large-scale chemical screening for pro- or anti-angiogenic molecules.

The present study aimed to investigate the angiogenic effects of novel chalcone-based derivatives in zebrafish *in vivo* model. Several chalcones were found to possess pro-angiogenic effect in wild type and transgenic line Tg(*fli1:egfp*) as evidenced by induction of ectopic subintestinal vein (SIV). Further investigation revealed that the expression of *vegfa*, an important angiogenic regulator, was upregulated on the treatment of these pro-angiogenic chalcone-based derivatives.

## 2. Results and Discussion

### 2.1. Chemistry

**Figure 1:**
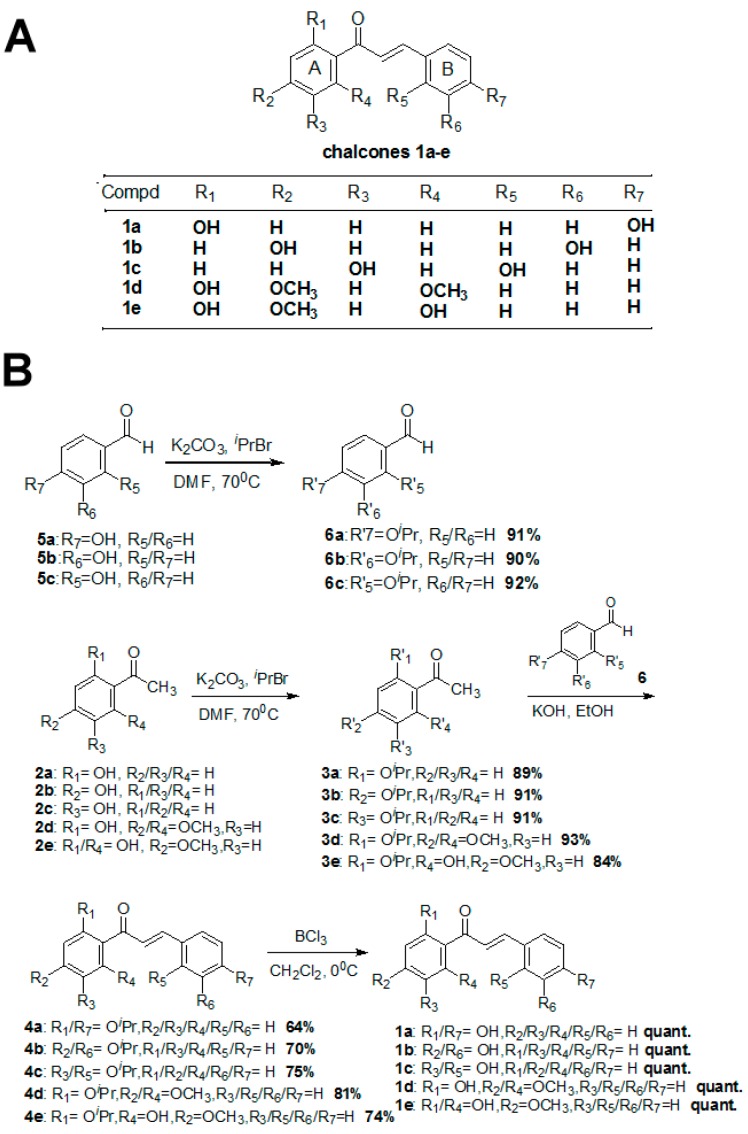
Structures and synthesis of chalcone and its derivatives. (**A**) Structures of chalcone and compound **1a**–**e**; (**B**) Synthesis of compounds **1a**–**e**.

For this study, we synthesized five chalcones **1a**, **1b**, **1c**, **1d** and **1e** (Figure 1A). Since it is possible that intramolecular hydrogen bonds observed in **1a**–**e** prevents aldol reactions, these aldol intermediates were obtained by using the similar procedure described previously [4,18,19,20]. Therefore, *O*-isoproxyacetophenones **3a**–**e** and *O*-isoproxybenzaldehydes **6a**–**c** were used as the starting material for our synthesis. The preparations of *O*-isoproxyacetophenones **3a**–**e** and *O*-isoproxybenzaldehydes **6a**–**c** were straightforward. Acetophenones **2a**–**e** and benzaldehydes **5a**–**c** were protected in excellent yield with isopropyl bromide and potassium carbonate in DMF. The isolated products **3a**–**e** were then reacted with appropriate benzaldehydes **6a**–**c** and 5N KOH to provide intermediates **4a**–**e** in 64%, 70%, 75%, 81% and 74% yields, respectively. The *O*-isopropyl ether was removed quantitatively with BCl_3_ to afford the target chalcones **1a**–**e** (Figure 1B), and each compound displayed >95% purity based on the ^1^H-NMR results. We were the first group to develop the methodology and protocols [4] to synthesize the target compounds **1a**–**e**. The intermediate compounds **2b**–**d**, **3b**–**d**, and **4b**–**d** reported in the present study are new and have not been reported before. Since we have previously reported the synthesis of **1a** and **1e**, the intermediate products (**2a**, **2e**, **3a**, **3e**, **4a**, and **4e**) are not considered to be new. In the present study, we employed the same methodology to synthesize the new compound **1b**, **1c**, and **1d**.

### 2.2. Chalcones Induced Pro-Angiogenic Effect on Zebrafish Embryos

During zebrafish embryonic development, angiogenesis is best characterized by formation of intersegmental vessels (ISV) and subintestinal veins (SIV). In zebrafish embryos, vascular development initiates at around 12 hour-post-fertilization (hpf), when hemangioblasts first appear along the lateral plate mesoderm. At 24 hpf, two axial vessels, the dorsal aorta (DA) and posterior cardinal vein (PCV), are first formed in the trunk of embryos. Meanwhile, bilateral ISVs sprout dorsally from the DA and finally reach the dorsal-most region of the somites and split dorsolaterally to form the dorsal longitudinal anastomotic vessels (DLAVs). The SIVs, originating from the common cardinal vein (duct of Cuvier) at 48 hpf, form in the shape of a basket on the dorsolateral surface of the yolk at 3 dpf [21]. In the presence of anti-angiogenic signal, defective ISV can be easily obsereved as judged by the shortened or no ISV formation. However, in the presence of pro-angiogenic stimulus, increased angiogenesis is not easily detected in ISV. Aberrant angiogenic sprouting appears until 3.5 dpf and can only be detecetd unambigiuosly by confocal microscopy [22]. On the contrary, observation of SIV development is suitable for detecting either anti- or pro-angiogenic compounds by simply calculating the number or length of SIV [23]. Additionally, SIV development can also be easily monitored in wild type embryos by the staining endogenous alkaline phosphatase activity, which is not present in early developing ISV.

**Figure 2:**
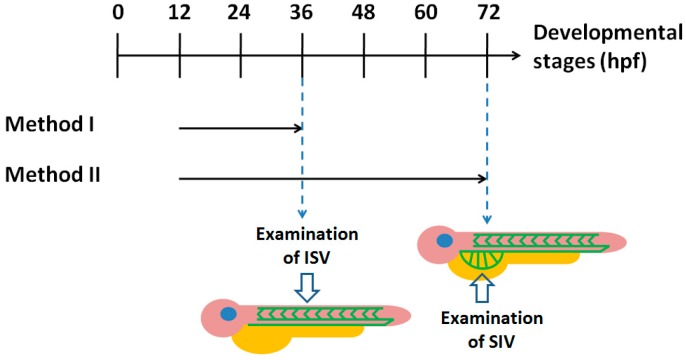
Exposure methods used in this study. Zebrafish wild type or Tg(*fli1:egfp*) embryos were treated with different concentration of chemicals from 12 to 36 hpf (method I) or from 12 to 72 hpf (method II). The embryos treated via method I were subjected to the analysis of intersegmental vessels (ISVs) formation, while those treated via method II were subjected to analysis of subintestinal veins (SIVs) formation.

**Figure 3:**
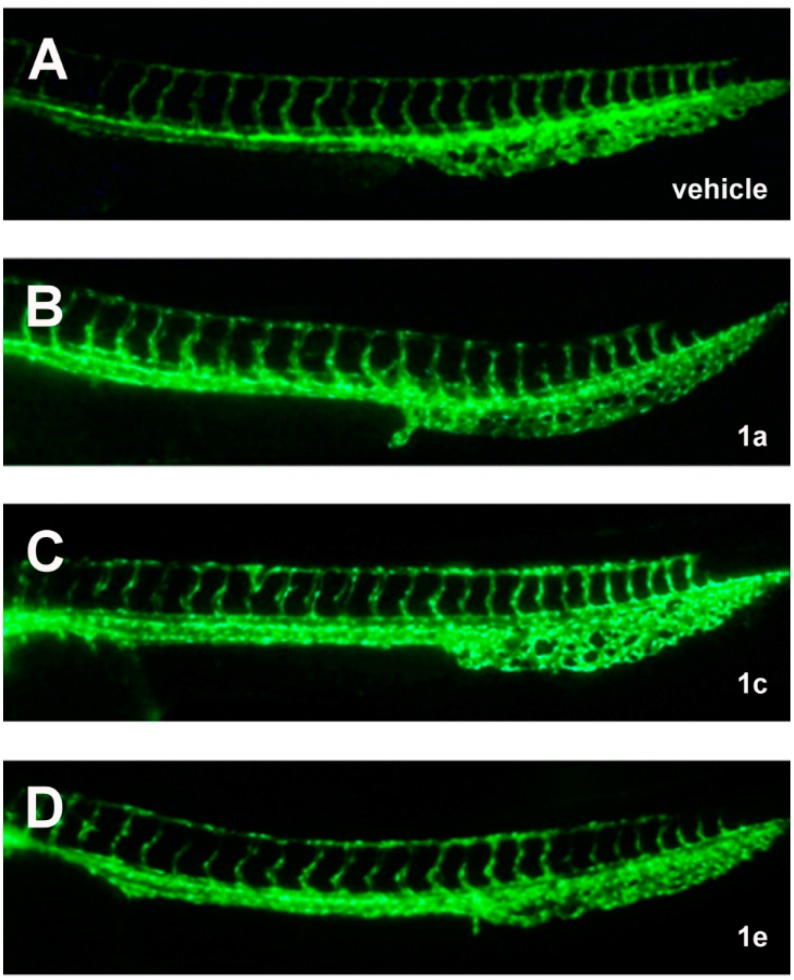
Chalcone derivatives had no effect on the formation of angiogenic intersegmental vessels (ISVs). (**A**–**D**) Representative images of zebrafish trunk ISVs treated with 3 ppm of chalcone and its derivatives (in 0.12% DMSO) via exposure method I. All figures are lateral views with dorsal to the top and anterior to the left. The fluorescent vessels of embryos were observed at 36 hpf. Vehicle: 0.12% DMSO only.

To examine the effect of chalcones on vascular development of zebrafish embryos, we first treated the transgenic Tg(*fli1:egfp*) embryos with chalcones via two exposure methods to examine the effect on ISV (method I, 12–36 hpf) or SIV development (method II, 12–72 hpf) (Figure 2). Treatment of chalcones with 1 or 3 ppm via exposure method I caused no significant change in the formation of ISVs (Figure 3 and data not shown), suggesting that chalcone derivatives had no effect on the initial development of trunk vasculature in zebrafish embryos.

**Figure 4:**
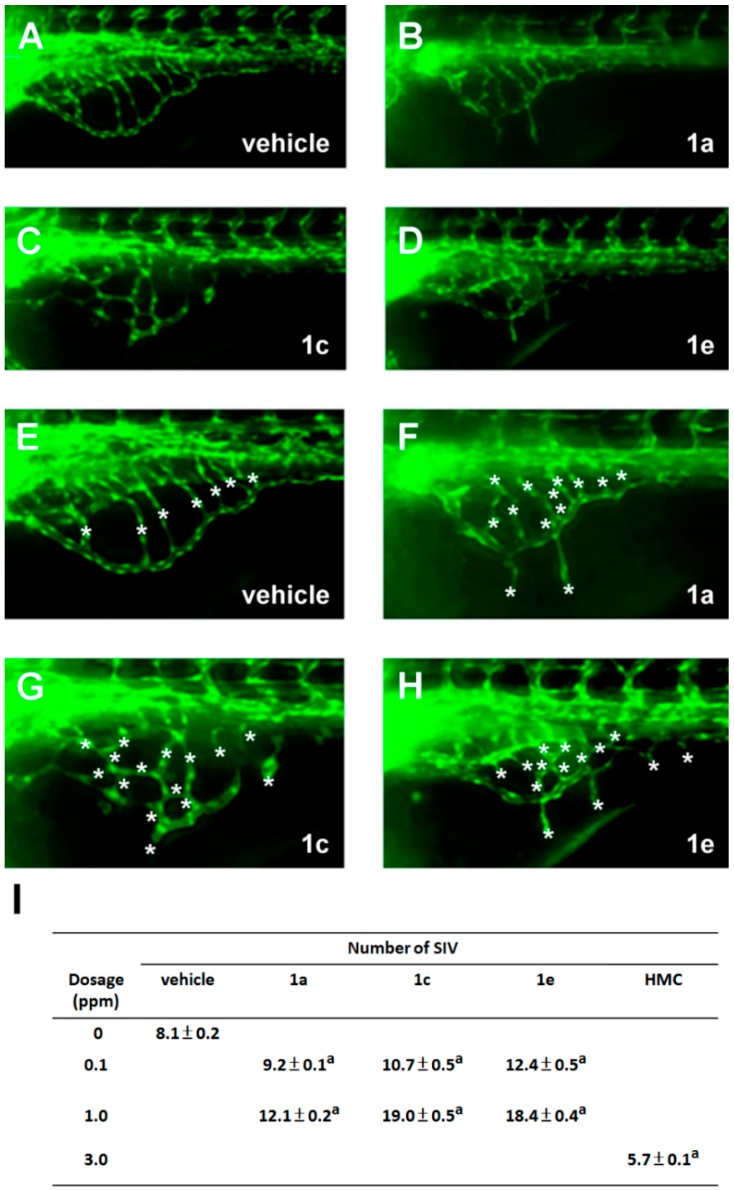
Chalcone derivatives caused pro-angiogenic effect on the zebrafish transgenic line Tg(*fli1:egfp*). (**A**–**D**) Representative images of fluorescent SIV basket of embryos treated with water containing 0.12% DMSO (vehicle) or chalcones derivatives (in 0.12% DMSO) via exposure method II. The fluorescent vessels of embryos were observed at 72 hpf; (**E**–**H**) Enlarged SIV regions in (**A**–**E**). The number of SIV was defined as the sum of the number of vessel within the SIV plus the number of angiogenic outgrowth sprouts (marked by asters); (**I**) Data are the mean ± S.D. of twenty embryos from three independent experiments (*n* = 60 in each group). A *t*-test with 0.05 of significance level, carried out by JMP statistical software (version 4.02), was used to assess the difference in the number of SIV between two groups. The result reported the number of SIV for all experiment groups are significantly different from vehicle group (**^a^**: *p* < 0.0001).

However, when transgenic zebrafish embryos were treated with chalcones via method II, some derivatives (compounds **1a**, **1c** and **1e**) induced abnormal patterning of the SIVs as evidenced by the additional branches of SIVs and the ectopic sprouting vessels outside the SIV basket (Figure 4A–D), suggesting that some chalcone compounds had pro-angiogenic activity on zebrafish embryos. Compound **1b** had no pro-angiogenic activity, whereas compound **1d** (1 ppm) was extremely toxic (no survival embryos were observed after compound **1d** treatment). The quantification of pro-angiogenic effect on SIVs was measured by counting the SIV numbers in the treated embryos (Figure 4E–H). Chalcones slightly increased the numbers of SIVs at 0.1 ppm when compared to the vehicle control group. When the concentration of chemicals was elevated to 1 ppm, the pro-angiogenic effect was more pronounced as evidenced by the 1.5–2.3 fold of increase in the numbers of SIVs. The result indicated that chalcone derivatives (compounds **1a**, **1c** and **1e**) induce neovascularization in zebrafish SIVs in a dose-dependent manner. Among them, compounds **1c** and **1e** were more potent than the other derivatives in inducing ectopic SIV vessels (Figure 4I). As 2′-hydroxy-4-methoxychalcone (HMC) had been reported to possess anti-angiogenic activity using cell culture and chicken chorioallantoic membrane assay [24], we next explored whether this chalcone derivative had similar effect in the zebrafish embryos. Treatment of HMC at 3 ppm via method II resulted in anti-angiogenic effect in zebrafish embryos as evidenced by the decreased numbers of SIVs compared with vehicle control (Figure 4I). The above result indicated that zebrafish SIVs as a reliable model system for angiogenic study.

**Figure 5:**
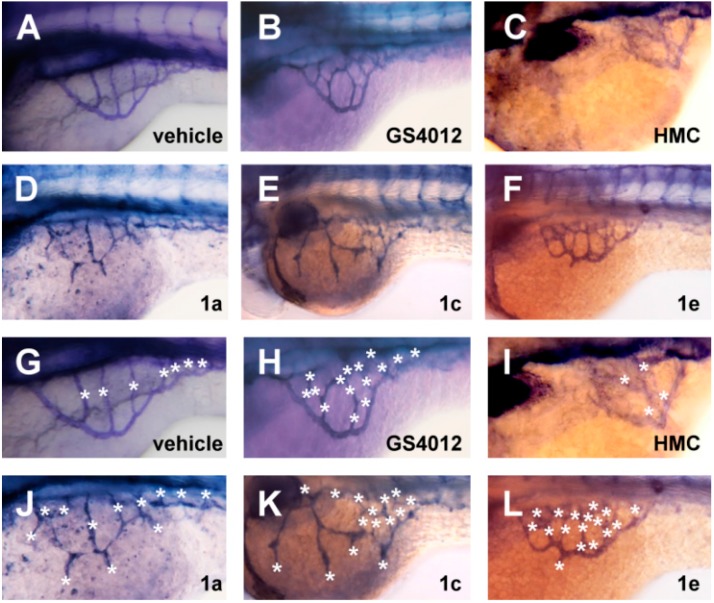
Chalcone derivatives caused pro-angiogenic effect on the wild type zebrafish. (**A**–**F**) Representative images of AP-stained SIV basket of embryos treated with GS4012, HMC, and chalcone derivatives via exposure method II; (**G**–**L**) Enlarged SIV regions in (**A**–**F**). The number of SIV was defined as the sum of the number of vessel within the SIV plus the number of angiogenic outgrowth sprouts (marked by asters). Embryos at 72 hpf were subjected to alkaline phosphatase staining.

To demonstrate whether the pro-angiogenic effect of chalcones can also be observed in wild type embryos, we treated the chemicals with wild type embryos via method II and visualized the SIV formation by endogenous alkaline phosphatase staining. GS4012, a pro-angiogenic compound, and HMC, the chalcone with anti-angiogenic activity, were included as positive and negative controls in the assay, respectively. As expected, GS4012 treatment led to increased numbers of SIV than vehicle group, whereas HCM treatment caused decreased numbers of SIV (Figure 5A–C). Similar to what was observed in transgenic Tg(*fli1:egfp*) embryos, increased numbers of the branch and ectopic SIV sprouting were also detected in chalcones-treated embryos when compared with vehicle control groups (Figure 5E–H). In addition, observation in wild type and Tg(*fli1:egfp)* transgenic embryos both showed that chalcone **1c** and **1e** exerts greater potent pro-angiogenic effects than **1a**. Taken together, chalcone derivatives had similar pro-angiogenic effect on both wild type and the transgenic embryos with fluorescent vessels.

### 2.3. Molecular Mechanism of Pro-Angiogenic Effects of Chalcone-Based Derivatives

**Figure 6:**
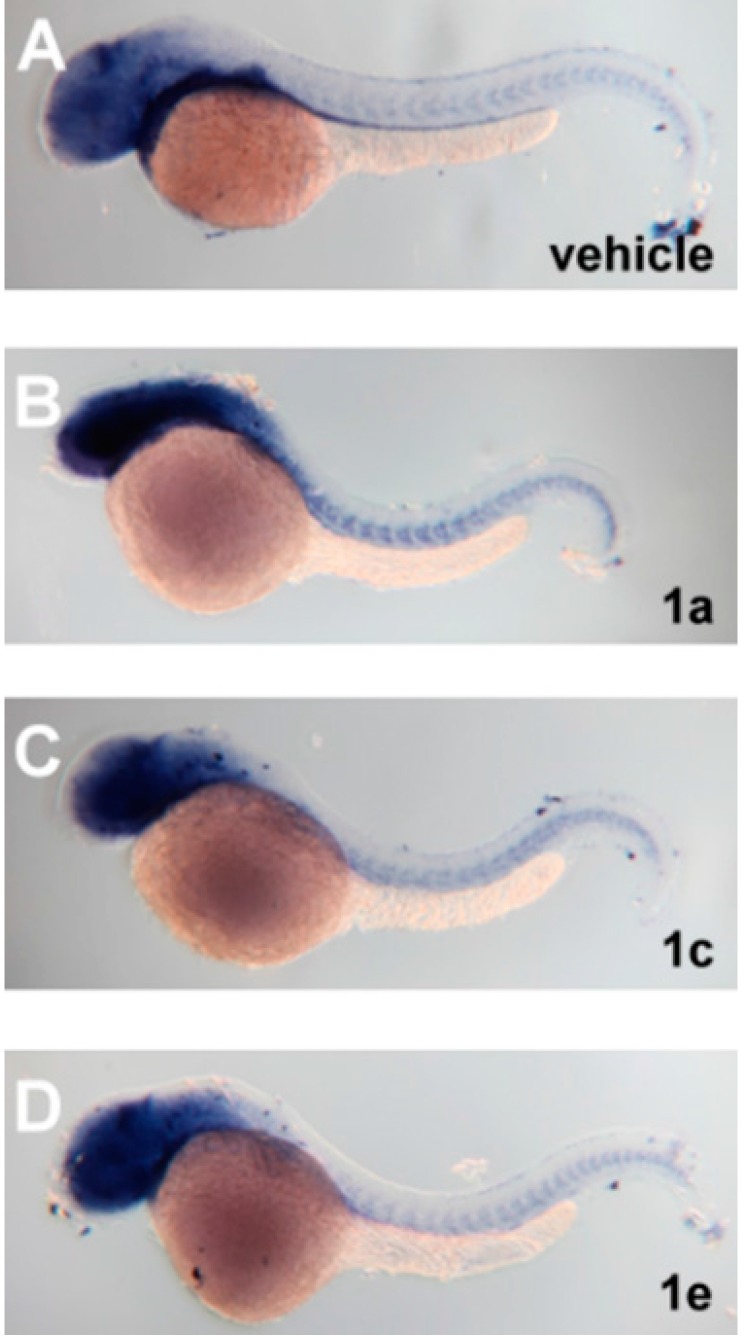
Effects of chalcone derivatives on the expression of *vegfaa*_165_.Whole-mount *in situ* hybridization was utilized to analyze the expression of *vegfaa*_165_ in zebrafish embryos treated with DMSO vehicle (**A**); 3 ppm of compound **1a** (**B**); **1c** (**C**) or **1e** (**D**) via exposure method I. Embryos at 36 hpf were subjected to whole-mount *in situ* hybridization. All figures were lateral views with anterior to the left and dorsal at the top.

To further explore the molecular mechanism of pro-angiogenic effects, we next investigated the effects of chalcone derivateson the expression of *vegfa*, the major ligand in VEGF signaling. In zebrafish, the *vegfa* gene is duplicated and the two genes are named *vegfaa* and *vegfab* [25]. Zebrafish *vegfaa* can produce two different isoforms: a 121-amino acid isoform (*vegfaa*_121_) and a 165-amino acid protein (*vegfaa*_165_). Previous studies showed that *vegfaa*_121_ is mainly involved in the formation of ISVs [26,27], whereas the *vegfaa*_165_ is primarily implicated in the formation of SIVs [27]. Due to the pro-angiogenic effect of SIVs on the treatment of chalcone-based derivatives, we investigated the expression of *vegfaa*_165_ using embryos treated with either compound **1a**, **1c** or **1e** through method I (12–36 hpf). By performing whole-mount *in situ* hybridization, we found that the mRNA levels of *vegfaa*_165_ in the chalcones-treated embryos were all upregulated as compared with the control groups (Figure 6). The expression of *vegfaa*_165_ was strongly enhanced in the anterior parts of embryos and moderately increased in adjacent somite areas along the trunk vascular regions (compare Figure 6B–D with 6A). Therefore, we speculated that the VEGF pathway might play a role in the pro-angiogenic effect of chalcone-based derivatives.

Cardiovascular and cerebrovascular diseases remain the main causes of death worldwide. These diseases are often associated with blockade or narrowing of the blood vessel network in the affected tissues or organs. Restoring blood supply in the affected regions is essential in the successful treatment of these ischemic diseases. Thus, compounds possessing pro-angiogenic activity are clinically important in therapeutic angiogenesis. Many natural and synthetic chalcone-based derivatives had been found to possess anti-angiogenic activities [8,22,28,29]. In the present study, we synthesized a series of novel chalcone-based derivatives and evaluated their angiogenic effect by zebrafish *in vivo* model. While the syntheses of compound **1a** and **1e** had been described in our previous report [4], the syntheses of **1b**, **1c**, and **1d** were first reported in the present study. Among them, chalcone derivatives, including compounds **1a**, **1c** and **1e** was found to induce ectopic SIV. The induction of ectopic blood vessels was accompanied by the enhanced expression of a key regulator of angiogenesis, *vegfa*. The exact mechanism of chalcone-based derivatives on the induction of *vegfa* expression is currently under investigation.

## 3. Experimental Section

### 3.1. Synthesis Chalcone Analogues

Melting points of the synthesized compounds were determined in open capillary tubes and are uncorrected. Proton NMR spectra were recorded at 300 MHz Varian Mercury-300 NMR spectrometer (Agilent, Santa Clara, CA, USA). Carbon NMR spectra were recorded at 75 MHz Varian Mercury-300 NMR spectrometer (Agilent). Proton and carbon chemical shifts are reported on the delta scale as parts per million (ppm) downfield from tetramethylsilane (TMS) as internal reference. Mass spectra were measured with a VG Analytical Model 70–250 s Mass Spectrometer, Varian, Palo Alto, CA, USA). All reagents were used as obtained commercially.

*3-(4-Hydroxyphenyl)-1-(2-hydroxyphenyl)-propenone* (**1a**): M.p. 164 °C (lit. [1] 157–158); ^1^H-NMR (CDCl_3_): 12.80 (1H, s, –OH), 10.80 (1H, s, –OH), 8.26 (1H, d, *J* = 8.0 Hz), 7.89 (1H, d, *J* = 15.6 Hz), 7.77 (1H, d, *J* = 15.6 Hz), 7.71 (2H, d, *J* = 8.4 Hz), 7.54 (1H, td, *J* = 8.1 Hz, 1.0 Hz), 7.01–6.95 (2H, m), 6.86 (2H, d, *J* = 8.4 Hz); ^13^C-NMR (CDCl_3_): 193.8, 163.5, 160.9, 145.4, 136.4, 134.5, 130.9, 125.8, 120.7, 119.3, 118.8, 117.9, 116.2; EI-MS *m*/*z* (rel.int.%): 240 (M^+^,100), 239 (63), 223 (33), 147 (17), 121 (55), 91 (94), 77 (28), 65 (33).

*3-(3-Hydroxyphenyl)-1-(4-hydroxyphenyl)-propenone* (**1b**): M.p. 259–260 °C (lit. [2] 261–262); ^1^H-NMR (*d*_6_-acetone): 9.40 (1H, s, –OH), 8.65 (1H, s, –OH), 8.07 (2H, d, *J* = 8.8 Hz), 7.77 (1H, d, *J* = 15.8 Hz), 7.65 (1H, d, *J* = 15.8 Hz), 7.29–7.21 (3H, m), 6.97 (2H, d, *J* = 8.8 Hz), 7.00–6.83 (1H, m); ^13^C-NMR (*d*_6_-acetone): 188.1, 162.8, 158.7, 143.9, 137.6, 131.9, 131.2, 130.8, 122.9, 120.8, 118.2, 116.2, 115.8; EI-MS *m*/*z* (rel.int.%): 240 (M^+^,100), 239 (63), 223 (33), 147 (17), 121 (55), 91 (16), 65 (29).

*3-(2-Hydroxyphenyl)-1-(3-hydroxyphenyl)-propenone* (**1c**): M.p. 173 °C (lit. [3] 173–174); ^1^H-NMR (*d*_6_-acetone): 9.32 (1H, s, –OH), 8.83 (1H, s, –OH), 8.15 (1H, d, *J* = 15.8 Hz), 7.82 (1H, d, *J* = 15.8 Hz), 7.78 (1H, dd, *J* = 7.6 Hz, 1.6 Hz), 7.61–7.53 (2H, m), 7.37 (1H, t, *J* = 8.0 Hz), 7.26 (1H, ddd, *J* = 8.0 Hz, 2.6 Hz, 1.6 Hz), 7.09 (1H, ddd, *J* = 8.0 Hz, 2.6 Hz, 1.6 Hz), 7.00–6.87 (2H, m); ^13^C-NMR (*d*_6_-acetone): 189.7, 158.0, 157.3, 140.3, 139.8, 131.9, 130.6, 129.3, 122.3, 122.0, 120.2, 120.0, 119.9, 116.5, 115.0; EI-MS *m*/*z* (rel.int.%): 240 (M^+^,72), 239 (100), 223 (91), 147 (45), 121 (23), 91 (27), 65 (22).

*1-(2-Hydroxy-4,6-dimethoxyphenyl)-3-phenylpropenone* (**1d**): M.p. 91–92 °C (lit. [4] 85–86); ^1^H-NMR (*d*_6_-acetone): 14.09 (1H, s, –OH), 8.88 (1H, d, *J* = 15.6 Hz), 7.75 (1H, d, *J* = 15.6 Hz), 7.62–7.57 (2H, m), 7.43–7.37 (3H, m), 6.10 (1H, d, *J* = 3.0 Hz), 5.96 (1H, d, *J* = 3.0 Hz), 3.90 (3H, s), 3.82 (3H, s); ^13^C-NMR (*d*_6_-acetone, 75 MHz): 192.5, 168.3, 166.1, 162.4, 142.2, 135.4, 129.9, 128.8, 128.2, 127.4, 106.2, 93.7, 91.1, 55.7, 55.4; EI-MS *m*/*z* (rel.int.%): 284 (M^+^,84), 283 (65), 207 (100), 181 (34), 103 (16), 77 (13).

*1-(2,6-Dihydroxy-4-methoxyphenyl)-3-phenylpropenone* (**1e**): M.p. 145 °C; ^1^H-NMR (*d*_6_-acetone): 12.07 (2H, s, –OH), 8.25 (1H, d, *J* = 15.6 Hz), 7.79 (1H, d, *J* = 15.6 Hz), 7.71–7.66 (2H, m), 7.46–7.40 (3H, m), 6.04 (2H, s), 3.81 (3H, s); ^13^C-NMR (*d*_6_-acetone): 193.4, 167.2, 165.4, 142.9, 136.4, 130.9, 129.8, 129.1, 128.3, 106.2, 94.6, 55.8; EI-MS *m*/*z* (rel.int.%): 270 (M^+^,6), 233 (15), 206 (22), 191 (23), 178 (100), 163 (26), 103 (45), 77 (59), 65 (24).

### 3.2. Fish Care

Mature zebrafish (wild type, WT; AB strain) and Tg(*fli1:egfp*) [17] were maintained at 28 °C with a photoperiod of 14 h light and 10 h dark, in an aquarium supplied with freshwater and aeration. Embryos were produced using standard procedures [30] and were staged according to standard criteria (hours postfertilization—hpf) or by days postfertilization (dpf) [31].

### 3.3. Chemical Treatment and Observation of Blood Vessels

Chemicals were treated to zebrafish embryos according to method I (12–36 hpf) or method II (12–72 hpf) described in Figure 2. Chalcone derivatives were dissolved in dimethylsulfoxide (DMSO) as stock solution (2500 ppm), and diluted to 3 ppm for treatment. For vehicle group, the final concentration of DMSO is 0.12%. To facilitate the visualization of blood vessels, phenylthiourea (PTU) was added into the media at a final concentration of 0.003% to inhibit melanization. At 36 or 72 hpf, photos of the ISVs or SIVs of the embryos were taken under a fluorescence microscope (Leica MZ95, Wetzlar, Germany) by a digital camera (Leica DFC 490).

### 3.4. Endogenous Alkaline Phosphatase Staining

Endogenous alkaline phosphatase activity was performed as described previously [32]. Briefly, the embryos were fixed with 4% paraformaldehyde at 4 °C overnight and permeated by acetone at −20 °C. After brief wash with PBST (phosphate buffer saline +0.1% Tween 20) and AP developing buffer (100 mM Tris·HCl, pH 9.5, 100 mM NaCl, 50 mM MgCl_2_), the embryos were developed with alkaline phosphatase substrate (0.34 mg/mL nitroblue tetrazolium, 0.18 mg/mL X-phosphate in 100 mM Tris pH 9.5, 100 mM NaCl, 50 mM MgCl_2_) and developed until desired signal was obtained. All embryos were observed and photographed as described in previous section.

### 3.5. Whole-Mount in Situ Hybridization

The procedures for whole-mount *in situ* hybridization was performed as previously described [33], except that *vegfaa*_165_ [34] were used as probes. Sequences encoding *vegfaa*_165_ isoform was amplified by PCR as previously described [34] and subsequently cloned into pGEM-T-easy vector. This plasmid was used to generate an antisense digoxigenin (DIG)-labeled riboprobe for *in situ* hybridization. Zebrafish embryos were fixed with 4% paraformaldehyde overnight and permeated by 100% methanol overnight at −20 °C. After that, embryos were rehydrated and digested with proteinase K. After a brief fixation by 4% paraformaldehyde, embryos were then prehybridized in hybridization buffer (50% formamide, 5 × SSC, 50 μg/mL yeast RNA, 25 μg/mL heparin) at 68 °C for at least 2 h and hybridized with DIG-labeled riboprobe overnight at 68 °C. Unbound and excessive probe was removed by washes in 2 × SSC followed by two washes in 0.2 × SSC for 30 min at 68 °C. After three rinses with PBST, embryos were blocked in 5% sheep serum (in PBST) for 1 h at room temperature and then incubated with sheep-anti-DIG-alkaline phosphatase (1:2000; Roche) for at least 2 h at room temperature. Unbound and excess antibody was removed by washing eight times in PBST for 2 h with gentle shaking. After three washes with AP developing buffer, the embryos were developed with AP substrate until desired signal was obtained. The reaction was stopped by washing in PBST for several times. The stained embryos were observed under a stereomicroscope or microscope equipped with DIC.

## 4. Conclusions

We have synthesized and identified several chalcone-based derivatives as a series of novel pro-angiogenic compounds using zebrafish *in vivo* model. Concomitant to the induction of angiogenesis, upregulated expression of *vegfa*, a key factor for angiogenesis, was observed. Further optimization of these chalcone derivatives may produce more potent pro-angiogenic agents for angiotherapy.
